# CD4 testing after initiation of antiretroviral therapy: Analysis of routine data from the South African HIV programme

**DOI:** 10.4102/sajhivmed.v21i1.1165

**Published:** 2020-12-14

**Authors:** Rivka R. Lilian, Natasha Davies, Louise Gilbert, James A. McIntyre, Helen E. Struthers, Kate Rees

**Affiliations:** 1 Anova Health Institute, Johannesburg, South Africa; 2 School of Public Health and Family Medicine, University of Cape Town, Cape Town, South Africa; 3 Division of Infectious Diseases and HIV Medicine, Department of Medicine, University of Cape Town, Cape Town, South Africa; 4 Department of Community Health, School of Public Health, University of the Witwatersrand, Johannesburg, South Africa

**Keywords:** CD4, HIV, South Africa, advanced clinical care, immunological non-responder, TIER.Net

## Abstract

**Background:**

People living with HIV (PLHIV) who have low CD4 counts require advanced clinical care (ACC) to minimise morbidity and mortality risk. These patients include immunological non-responders (INRs) with low CD4 counts despite a suppressed viral load.

**Objectives:**

To determine the proportion of patients with low CD4 counts after antiretroviral therapy (ART) initiation and to describe INRs within that group.

**Methods:**

Routine Three Interlinked Electronic Registers.Net (TIER.Net) data from four South African districts were analysed for adult PLHIV on ART > 12 months. Immunological non-responders were defined as patients on ART > 4 years who were virally suppressed (viral load < 1000 copies/mL) with a CD4 count ≤ 350 cell/mm^3^.

**Results:**

Baseline CD4 was recorded for 80.9% of the 869 571 patients newly initiating ART, with 37.2% of those starting ART since 2017 having baseline counts ≤ 200 cells/mm^3^. Amongst all 1 178 190 patients on ART, only 46.5% had a CD4 test after ART initiation and of these, 14.3% had CD4 ≤ 200 cells/mm^3^. This proportion was highest amongst patients on ART ≤ 2 years (19.7%) (*p* < 0.001). Amongst virally suppressed patients, 20.0% were INRs. Immunological non-response was significantly more likely amongst patients on second-line ART (adjusted odds ratio [aOR] 1.79), those aged 35-45 and ≥ 45 years (aOR 1.15 and 1.50, respectively), males (aOR 2.28) and patients with confirmed TB (aOR 2.49), and was significantly less likely in cases with higher baseline CD4 count (aOR 0.35).

**Conclusion:**

CD4 testing subsequent to ART initiation is poorly implemented and there is a notable proportion of patients with low CD4 counts. Guidelines regarding CD4 testing and ACC need to be more widely implemented to identify patients with low CD4 counts and improve their outcomes.

## Introduction

Antiretroviral therapy (ART) improves outcomes of people living with human immunodeficiency virus (PLHIV) by reducing HIV viral load (VL), which enables immune recovery, including recovery of CD4 count. Where patients have CD4 counts below 200 cells/mm^3^, known as advanced HIV disease, advanced clinical care (ACC) should be provided to improve patient outcomes, including screening, management of opportunistic infections and focussed adherence support.^1^ Advanced clinical care is particularly important in South Africa, considering that up to 40% of adults initiate ART with CD4 < 200 cells/mm^3^.^2^ Furthermore, up to 50% of patients receiving ART demonstrate poor CD4 recovery, dependant on multiple factors including baseline CD4 count and CD4 recovery definition.^3,4,5,6^ In some patients, known as immunological non-responders (INRs), CD4 count remains low despite a suppressed VL, which significantly increases the risk of mortality.^7^

South African guidelines include baseline CD4 testing for ART patients to assess cotrimoxazole prophylaxis (CPT) eligibility (where CD4 count is ≤ 200 cells/mm^3^) and to determine susceptibility to opportunistic infections.^8^ CD4 monitoring should be repeated 12 months after ART initiation, 6 monthly until CPT eligibility ceases, 6 monthly if VL increases above 1000 copies/mL and if a patient requires re-initiation onto ART.^8^ Such CD4 testing is critical to ensure correct ACC management for patients with low CD4 counts to mitigate higher morbidity and mortality risks.

The South African ART programme is routinely managed using an electronic database known as Three Interlinked Electronic Registers.Net (TIER.Net). This study analysed routine programmatic TIER.Net data to assess implementation of post-ART CD4 testing and occurrence of poor CD4 recovery in order to highlight areas for intervention to improve CD4 monitoring and subsequent patient management. Specifically, this study aimed to: (1) calculate the proportion of patients on ART with CD4 tests subsequent to ART initiation, (2) describe the proportion of patients with subsequent CD4 counts ≤ 200 and ≤ 350 cells/mm^3^ by ART duration and (3) assess the proportion of INRs and describe their characteristics.

## Methods

### Data source and study population

Three Interlinked Electronic Registers.Net data were extracted in March 2020 for Johannesburg and Sedibeng districts in Gauteng province and Capricorn and Mopani districts in Limpopo province. These districts were chosen as a convenience sample, as Anova Health Institute is the designated United States Agency for International Development support partner in these districts. Urban Johannesburg district is very densely populated, with 3162.1 persons/km^2^, compared with 236.0, 61.7 and 61.2 persons/km^2^ in Sedibeng, Capricorn and Mopani, respectively.^9^ Medical scheme coverage is highest in Johannesburg (22.2%), followed by Sedibeng (20.8%), Capricorn (8.3%) and Mopani (6.8%).^9^ Antenatal HIV prevalence, an indicator of overall population prevalence, was 34.1% in Sedibeng, 30.9% in Johannesburg, 26.6% in Mopani and 22.5% in Capricorn in 2017.^10^

Records from TIER.Net were included in the analysis for adult PLHIV aged 15–80 years who had initiated ART from 2004 onwards and had been on ART for a minimum of 12 months (*n* = 1 224 366). Records were excluded where there were data quality concerns regarding CD4 testing, namely counts ≤ 0 or ≥ 2000 cells/mm^3^ and nonsensical testing dates (*n* = 722). Records with nonsensical VL testing dates were also excluded (*n* = 9), as were records from facilities which had not exclusively used TIER.Net as their ART management tool, resulting in incomplete TIER.Net data (*n* = 45 445). The final data set comprised 1 178 190 records – 673 606 from Johannesburg, 160 607 from Sedibeng, 162 020 from Capricorn and 181 957 from Mopani.

### Statistical analysis

The proportion of patients with a baseline CD4 test and CD4 test subsequent to baseline was calculated, the former was for patients newly initiating ART and the latter was for all patients in the cohort (both new initiators and re-initiators). Post-baseline CD4 tests were defined as CD4 tests performed more than 3 months after ART where the CD4 count was not a duplicate of the baseline count. The proportion of patients with post-baseline CD4 counts ≤ 200 and ≤ 350 cells/mm^3^ was calculated and compared between different ART durations using a chi-squared test.

Immunological non-responders were defined as patients who had been on ART for more than 4 years and were virally suppressed (VL < 1000 copies/mL) with a CD4 count ≤ 350 cell/mm^3^ based on CD4 testing performed 1 year before to 2 years after the VL test. Time on ART for all patients, including those receiving second- or third-line ART regimens, was calculated as overall time on treatment. Mixed effects logistic regression was used to assess characteristics of INRs, adjusting for random effects at the district level. Fixed effects covariates included ART regimen (first-line, second-line or third-line), age at ART start, gender, baseline CD4 and tuberculosis (TB) status. *p* < 0.05 was considered significant.

### Ethical consideration

The study was approved by the Human Sciences Research Council Research Ethics Committee (REC 3/22/08/18). Individual patient consent was not required, as no data were collected for the purposes of this study. Anonymised TIER.Net data that were routinely collected at healthcare facilities for monitoring purposes were used.

## Results

### Implementation of CD4 testing

Baseline CD4 count was recorded for 80.9% of the 869 571 patients newly initiating ART. Amongst all 1 178 190 adults on ART, only 46.5% had a CD4 count recorded subsequent to baseline. Amongst patients who had been on ART for 12–18 months (*n* = 56 181), only 21.9% had a post-baseline CD4 test on record.

### Low CD4 counts

Amongst all patients with a baseline CD4 (*n* = 703 869), 50.3% had counts ≤ 200 cells/mm^3^ and amongst those starting ART since 2017, 37.2% had a baseline count ≤ 200 cells/mm^3^. CD4 count decreased after baseline in 11.0% of the 443 443 patients with both a baseline and subsequent CD4. Amongst all patients with a CD4 test performed after ART start, 14.3% (*n* = 78 494) had a CD4 count ≤ 200 cells/mm^3^. This proportion was highest amongst patients on ART ≤ 2 years (19.7%) compared with longer treatment durations (14.8%, 14.2% and 13.8% amongst patients on ART 2–4, 4–6 and > 6 years, respectively, *p* < 0.001).

Just over one-third of patients with a post-baseline CD4 test had a CD4 count ≤ 350 cells/mm^3^ (35.5%, *n* = 194 140). This proportion was highest amongst patients on ART ≤ 2 years (43.9%) compared with longer durations (36.1%, 35.2% and 34.7% amongst those on ART 2–4, 4–6, and > 6 years, respectively, *p* < 0.001).

### Immunological non-responders

Amongst virally suppressed patients on ART for more than 4 years, 20.0% (*n* = 18 556) were INRs. Median CD4 count amongst INRs was 259 cell/mm^3^ (interquartile range 127), with 29.5% having a CD4 count ≤ 200 cells/mm^3^. Immunological non-response was significantly more likely amongst second- or third-line ART patients compared with first-line (odds ratio [OR] 1.85 and 1.54, respectively), amongst older patients compared with those aged 15–25 years (OR 1.12, 1.49 and 1.93 in patients aged 25–35, 35–45 and ≥ 45 years, respectively), in males (OR 2.45) and amongst patients with TB (OR 3.57; Table 1). The odds of immunological non-response were significantly lower amongst patients with baseline CD4 > 350 cells/mm^3^ compared with CD4 ≤ 350 (OR 0.32). In multivariate analysis, INR remained significantly more likely amongst patients on second-line ART (adjusted OR [aOR] 1.79), those aged 35–45 and ≥ 45 years (aOR 1.15 and 1.50, respectively), males (aOR 2.28) and patients with confirmed TB (aOR 2.49). The odds of INR also remained significantly less likely amongst patients with higher baseline CD4 (aOR 0.35).

**TABLE 1:** Characteristics of immunological non-responders (CD4 count ≤ 350 cells/mm^3^) compared with immunological responders (CD4 count > 350 cells/mm^3^) amongst patients with a suppressed viral load who had been on ART for more than 4 years.

Variables	Immunological non-responder, *n* = 18 556		Immunological responder, *n* = 74 134		Unadjusted OR		Adjusted OR†	
	*n*	%	*n*	%	OR (95% CI)	*p*-value	OR (95% CI)	*p*
**Last ART regimen**								
First line	13 615	18.3	60 709	81.7	Ref		Ref	
Second line	2208	29.5	5282	70.5	**1.85 (1.76–1.96)**	**< 0.001**	**1.79 (1.68–1.91)**	**< 0.001**
Third line	34	26.6	94	73.4	**1.54 (1.04–2.28)**	**0.031**	1.41 (0.85–2.35)	0.188
Unassigned	22	22.5	76	77.6	1.22 (0.76–1.97)	0.403	0.83 (0.44–1.59)	0.581
**Age at ART start, years**								
15–25	1104	15.4	6073	84.6	Ref		Ref	
25–35	5836	16.9	28 748	83.1	**1.12 (1.04–1.20)**	**0.001**	0.99 (0.90–1.09)	0.886
35–45	6662	21.1	24 851	78.9	**1.49 (1.39–1.60)**	**< 0.001**	**1.15 (1.05–1.27)**	**0.003**
≥ 45	4954	25.5	14 462	74.5	**1.93 (1.80–2.08)**	**< 0.001**	**1.50 (1.37–1.66)**	**< 0.001**
**Gender**								
Female	9902	15.3	54 749	84.7	Ref		Ref	
Male	8654	30.9	19 385	69.1	**2.45 (2.37–2.53)**	**< 0.001**	**2.28 (2.19–2.38)**	**< 0.001**
**Baseline CD4, cells/mm^3^**								
≤ 350	13 831	21.6	50 266	78.4	Ref		Ref	
> 350	664	8.0	7669	92.0	**0.32 (0.29–0.34)**	**< 0.001**	**0.35 (0.32–0.38)**	**< 0.001**
**TB status**								
None/unknown	18 393	19.9	73 955	80.1	Ref		Ref	
Confirmed TB	163	47.7	179	52.3	**3.57 (2.88–4.42)**	**< 0.001**	**2.49 (1.81–3.43)**	**< 0.001**

ART, antiretroviral therapy; CI, confidence interval; OR, odds ratio; Ref, reference; TB, tuberculosis.

Data are *n* (%). Total value differs between variables because of missing data.

Statistically significant differences are shown in bold.

† , Mixed effects logistic regression for characteristics of immunological non-responders, adjusting for random effects at the district level. Fixed effects covariates include ART regimen level, age at ART start, gender, baseline CD4 count and TB status.

## Discussion

This study of routine data from four South African districts demonstrates poor implementation of CD4 testing subsequent to ART initiation and a notable proportion of patients with low CD4 counts even after initiating ART. Amongst patients on ART for 12–18 months who should have received a routine 12-month CD4 test according to national guidelines,^8^ less than one quarter had a post-baseline CD4 test recorded. Considering that amongst those who did access CD4 testing, CD4 count was below 200 cells/mm^3^ in 20% of patients on ART ≤ 2 years, a considerable number of patients with low CD4 counts are being missed. These patients would not receive ACC interventions, including CPT, which may contribute to higher morbidity and mortality.

Immunological non-response was found in 20.0% of virally suppressed patients in this cohort, in line with reported rates of 10% – 40%.^3,5,7^ There is an ongoing need for CD4 count monitoring subsequent to ART initiation in order to identify INRs who require extra clinical care. Efforts should focus on older patients, males and those starting ART with low baseline CD4, as has been previously demonstrated,^3,4,5,6,7^ as well as PLHIV with TB coinfection. It is concerning that low baseline CD4 is significantly associated with immunological non-response, as a notable proportion of patients initiating ART in South Africa do so with low CD4 counts.^2^ In particular, older patients and males are more likely to present late for HIV care with low baseline CD4,^2^ which may contribute to the poor CD4 recovery of these patients. Continued engagement with communities, particularly with men and older clients, is required to emphasise the importance of engaging with ART services before CD4 counts have dropped substantially. The association between INR and second- or third-line regimens may be because of prolonged viral non-suppression associated with treatment failure. It is possible that low CD4 counts are a result of delayed switching from a failing to effective regimen, which would need to be further investigated.

The strength of this study is the large sample size from a routine patient-level data set that includes clinical variables. However, there are a number of limitations. Firstly, a post-baseline CD4 test was missing in more than half the cohort, which may have introduced bias into the estimation of INRs. This estimation may also have been somewhat inflated by the inclusion of CD4 tests performed up to 2 years after the VL test. Secondly, we did not have access to all post-baseline CD4 and VL tests; only the most recent result was available. We could, therefore, not assess change in CD4 count over time following ART initiation, nor could we assess timing of viral suppression amongst patients with treatment failure who were switched to a second- or third-line regimen, which may have impacted time to CD4 recovery. Thirdly, we only had access to CD4 test results that had been captured into TIER.Net. CD4 counts that were missing from TIER.Net could, therefore, not be included in the analysis. It is unclear whether missing CD4 data represent poor implementation of CD4 testing in clinical practice or a data capturing problem, and this warrants further investigation. Finally, although we believe the study sample to be generally representative of the South African population as it includes both urban and rural areas, as well as districts with high and low socio-economic status, findings should be extrapolated to other areas with caution, particularly because this was not a random sample.

In conclusion, it is essential that healthcare workers are educated regarding the ongoing importance of correctly implementing CD4 testing guidelines in the universal test and treat era to identify patients with low CD4 counts who require extra clinical care. Quality of care needs to be improved and existing ACC guidelines more widely implemented in order to support improved clinical outcomes in patients with low CD4 counts.
